# Latest updates on the serotonergic system in depression and anxiety

**DOI:** 10.3389/fnsyn.2023.1124112

**Published:** 2023-05-09

**Authors:** Jianwen Lin, Wenxin Liu, Jing Guan, Jianing Cui, Ruolin Shi, Lu Wang, Dong Chen, Yi Liu

**Affiliations:** ^1^Department of Neurology, Dalian Municipal Central Hospital, Central Hospital of Dalian University of Technology, Dalian, China; ^2^Department of Graduate Studies, Dalian Medical University, Dalian, China; ^3^Department of Pediatrics, Yingkou Economic and Technological Development Zone Central Hospital, Yingkou, China; ^4^Department of Neurosurgery, Dalian Municipal Central Hospital, Central Hospital of Dalian University of Technology, Dalian, China

**Keywords:** depression, anxiety, therapeutic target, 5-hydroxytryptamine, serotonin receptor, anxiolytic, anti-depressant

## Abstract

Psychiatric disorders are among the leading causes of global health burden, with depression and anxiety being the most disabling subtypes. The two common disorders, depression and anxiety, usually coexist and are pathologically polygenic with complicated etiologies. Current drug-based therapies include selective serotonin reuptake inhibitors, serotonin and norepinephrine reuptake inhibitors, and 5-hydroxytryptamine partial agonists. However, these modalities share common limitations, such as slow onset and low efficacy, which is why potential mechanistic insights for new drug targets are needed. In this review, we summarize recent advances in brain localization, pathology, and therapeutic mechanisms of the serotonergic system in depression and anxiety.

## 1. Introduction

Psychiatric disorders are among the leading causes of global health burden, with depression and anxiety being the most disabling subtypes. Depression and anxiety usually coexist, and their etiologies are complex and polygenic in nature (García-Gutiérrez et al., 2020). The serotonergic hypothesis, proposed by Professor Coppen in 1967, suggests that 5-hydroxytryptamine (5-HT) deficiency is the main underlying cause of depression and anxiety, a notion that was further supported by numerous studies. In the central nervous system (CNS), 5-HT is mainly secreted from the midbrain and pontine nuclei, including the dorsal raphe, medial and rostral nuclei, caudal nucleus, interpeduncular nucleus, B9 5-HTergic cell group, and reticular structures. Serotonin signaling occurs in most parts of the brain, including the prefrontal, frontal, parietal, and occipital cortices, cingulate, septum, hippocampus, parahippocampal gyrus, amygdala, entorhinal cortex, neostriatum, ventral striatum, substantia nigra, ventral tegmental area, thalamus, hypothalamus, locus coeruleus, and papillary nucleus (Carr and Lucki, 2011). Different neurocircuits employ different serotonergic receptors to produce unique neurobiological effects, many of which have been the target of therapeutic drug design. In this review, we summarize recent findings in the cerebral localization of serotonin receptors and the pathological and therapeutic mechanisms of the serotonergic system in depression and anxiety.

## 2. Therapeutic advancement in depression and anxiety

The serotonergic hypothesis is considered the main mechanism underlying depression and anxiety (Gelenberg, 2010). The first generation of antidepressants are the monoamine oxidase inhibitors, which improve mood and stimulate activity; however, their use is limited due to their side effects of hepatic toxicity and hypertensive crises. Tricyclic antidepressants (TCAs) such as imipramine and clomipramine were subsequently developed and worked mainly by blocking the reuptake of 5-HT and norepinephrine (Cipriani et al., 2018). Due to their non-specificity and side effects, TCAs are currently prescribed for cases of refractory depression. In the late 1980s, second-generation antidepressants were developed including SSRIs, serotonin norepinephrine reuptake inhibitors (SNRIs), specific serotonergic antidepressants, and other related drugs. SSRIs such as escitalopram, fluoxetine, and sertraline are the most commonly prescribed drugs for depression and anxiety and are the first-line drugs for major depressive disorder owing to their tolerability, efficacy, and favorable side effect profile (Lanfumey and Hamon, 2004). Notably, fluoxetine may be the optimal choice for children and adolescents with depression and anxiety (Cipriani et al., 2016; Davey et al., 2019). Additionally, SSRIs are used in the treatment of generalized anxiety disorder, panic disorder, and obsessive-compulsive disorder (Healy et al., 2019). However, they have the shortcomings of slow onset and compromised effectiveness, and approximately 70% of patients respond incompletely to SSRIs, and the remaining patients are difficult to treat (Martiny, 2017).

In recent years, new therapeutics have been developed including non-competitive NMDA receptor antagonists (Mamdani et al., 2014), anticholinergic drugs (Mendez-David et al., 2014), opioid modulators (Samuels et al., 2017; Browne et al., 2020), and new 5-HT receptor subtype modulators. The high selectivity of 5-HT receptor agonists or antagonists potentially overcomes the main shortcomings of conventional serotonergic therapeutics, namely, low efficacy and slow onset. For example, the selective 5-HT4 receptor agonist RS67333 was reported to ameliorate depression-like behavior in a mouse model with depression/anxiety within 10–14 days, which was faster than the duration of conventional antidepressants (Mendez-David et al., 2014). Moreover, classifying patients with mood disorders according to their pathophysiological mechanisms may provide more targeted and effective therapeutics. Additionally, genetic factors such as single nucleotide polymorphisms in the 5-HT1A promoter, rs6295, and C-1019G are related to the density and activity of 5-HT1A receptors and contribute to the susceptibility to psychiatric disorders and may aid in guiding therapeutics (Razakarivony et al., 2021).

## 3. Serotonin and its receptors

**Figure 1:**
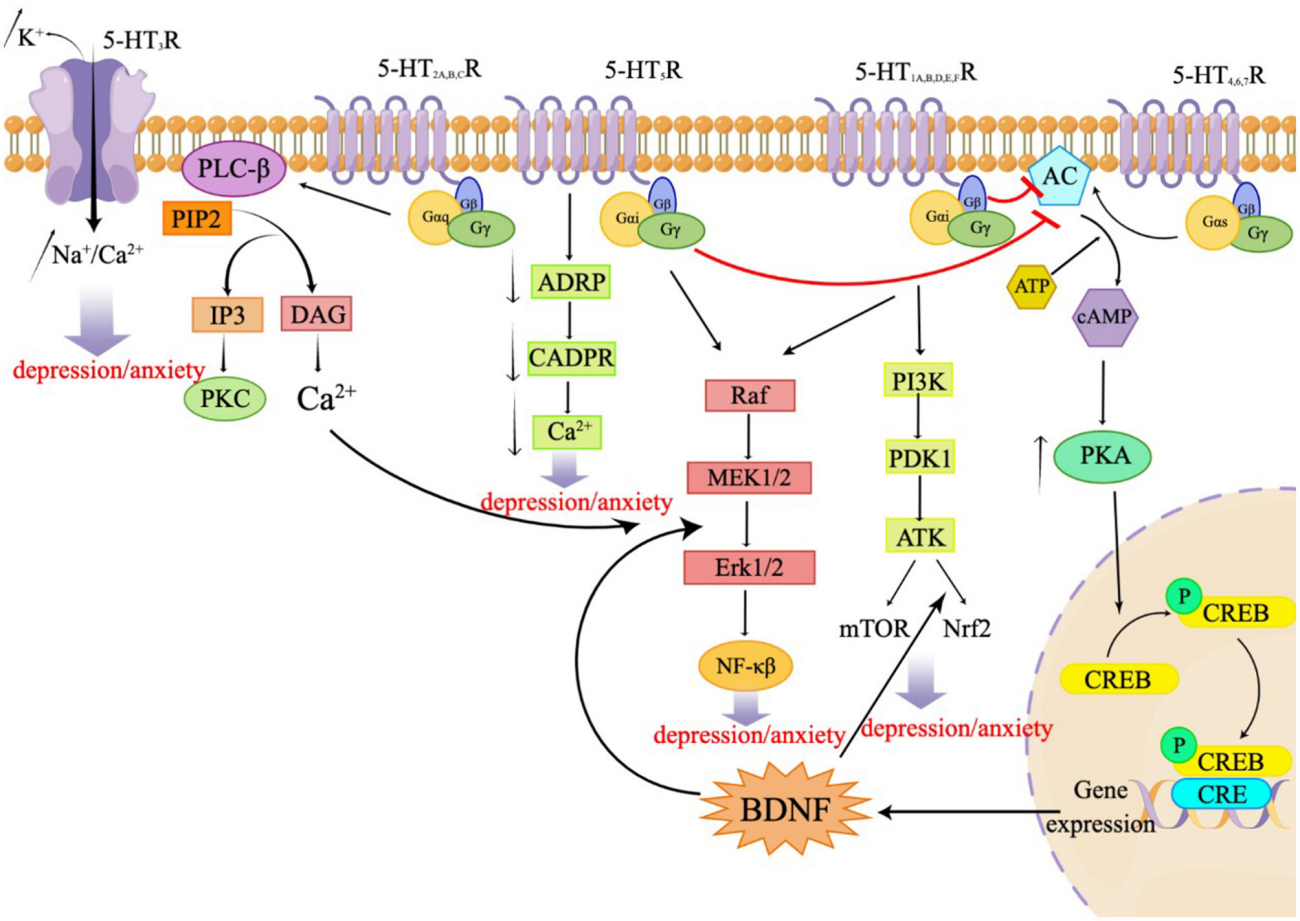
The signal transduction pathways of the associated receptors in anxiety and depression.

Serotonin, also known as 5-HT, is an important neurotransmitter in the CNS. Serotonin is implicated in several physiological phenomena, including the regulation of body temperature, appetite, sleep cycle, blood pressure, mood, and pain. 5-HT is mostly released by serotonergic neurons located in the brain stem and acts by binding to specific receptors. A total of seven families of 5-HT receptors (5-HT1 through 5-HT7) and at least 14 different subtypes have been identified so far (Nichols and Nichols, 2008; Muit et al., 2022). All 5-HT receptors belong to the G protein-coupled receptor (GPCR) superfamily, except the 5-HT3 receptor, which is a ligand-gated ion channel. These receptors are either presynaptic or postsynaptic and serve different functions. Presynaptic 5-HT receptors are autoreceptors, which monitor the extracellular 5-HT concentrations to regulate the 5-HT release and signal transduction. Postsynaptic 5-HT receptors translate the chemical signals to the postsynaptic neurons to regulate multiple neural circuits. Almost all 5-HT receptor subtypes are implicated in depression and/or anxiety through specific mechanisms (Yohn et al., 2017). The 5-HT1A, B, D, E, and F receptors and the 5-HT5 receptor are Gi/o-coupled receptors that inhibit the production of AC and cAMP, resulting in a reduction in the intracellular cAMP level. These receptors activate the PI3K-Akt signaling pathway, regulating mTOR and Nrf2, mediating the ERK/MAPK signaling pathway, and participating in the ERK1/2 pathway to modulate NF-kB. This, in turn, influences synaptic plasticity and behaviors related to depression in neurons (Albert and Vahid-Ansari, 2019; Barnes et al., 2021; Calhoun et al., 2023). The 5-HT2A, B, and C receptors are Gq-coupled receptors that activate phospholipase C (PLC), leading to an accumulation of inositol triphosphate (IP3) and activation of protein kinase C (PKC), resulting in an increase in intracellular calcium ion concentration. This subsequent activation of ERK1/2 is involved in the regulation of synaptic plasticity and plays a role in various physiological and pathological processes. The 5-HT3 receptor functions as a ligand-gated ion channel, allowing the permeation of Na+, K+, and Ca2+ ions. Notably, Ca^2+^ concentration dependently decreases the Na+ current induced by 5-HT. The 5-HT4, 6, and 7 receptors are Gs-coupled receptors that activate adenylate cyclase, thereby stimulating the formation of cyclic adenosine monophosphate (cAMP) (Barnes et al.). The generated cAMP, in turn, activates protein kinase A (PKA), leading to an increase in the activation of cyclic AMP response element binding protein (CREB) and the expression of brain-derived neurotrophic factor (BDNF) (Wang, 2021). The 5-HT5R can stimulate the release of Ca^2+^ by coupling uniquely with adenylate cyclase and inhibiting the formation of cyclic ADP ribose (cADPR). Additionally, this receptor mediates the extracellular signal-regulated kinase 1/2 (ERK1/2) signaling pathway, which promotes the synthesis of BDNF (Gerasimenko et al., 2020; Lu et al., 2022). The following sections outline the different 5-HT receptors and their role in anxiety and depression. Figure 1 presents the signal transduction pathways of the related receptors, 5-HT1 receptors.

### 3.1. 5-HT1A receptor

**Figure 2:**
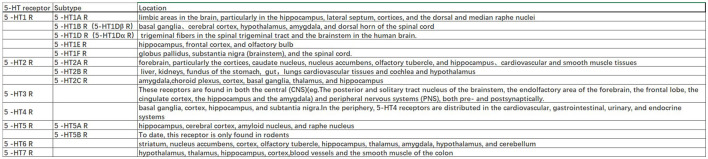
Location of serotonergic receptors.

The 5-HT1A autoreceptor is a key negative regulator of 5-HT activity. The Htr1a gene encoding the 5-HT1A receptor blocks the function of specific repressors, such as Hes1, Hes5, Deaf1, Freud-1, and Freud-2, promoting upregulation of the 5-HT1A autoreceptor and increasing its expression in 5-HT1A neuronal cells. 5-HT1A receptors are widely distributed in the CNS, cardiovascular system, and gastrointestinal system (Figure 2) and play pivotal roles in multiple pathophysiological processes (Guzel and Mirowska-Guzel, 2022). In the CNS, 5-HT1A receptors are mainly located in the hippocampus, nasal septum, amygdala, and cortical limbus and act as both autoreceptors and heteroreceptors, which makes it a therapeutic target for depression, anxiety, schizophrenia, pain, cognitive impairment, and neurodegenerative diseases. Several 5-HT1A-based drugs have been registered and approved, including antidepressants and anti-migraine drugs (Guzel and Mirowska-Guzel, 2022). In a clinical study assessing the therapeutic effect of targeting both autoreceptor and heteroreceptor functions of the 5-HT1A receptor, Ping Dolor, a ß-adrenoceptor/5-HT1A receptor antagonist, failed to improve the selective serotonin reuptake inhibitor (SSRI)-resistant depression. On the other hand, full or partial agonists of the 5-HT1A receptors such as azapirones, ixabepilone, buspirone, and gefitinib, showed both anxiolytic and antidepressive effects. Furthermore, two new antidepressants, vilazodone and vortioxetine (Slifirski et al., 2021) (Table 1), appear to overcome the shortcomings encountered with SSRIs and partial 5-HT1A agonists, including delayed effect onset and treatment resistance. Finally, Cannabidiol (Table 1), one of the main compounds in cannabis, has therapeutic potential against anxiety, depression, psychosis, epilepsy, and neuroprotection through the regulation of 5-HT1A (García-Gutiérrez et al., 2020).

**Table 1:** Drugs and their associated targets.

**Drug**	**Pharmacological targets**	**Intervention**	**Clinical efficacy**	**Safety profile**
Vilazodone	Inhibitor of the serotonin transporter and partial agonist of 5-HT1A	Double-blind randomized placebo-controlled studies	Effective drug for patients with MDD	High rate of gastrointestinal side effects and insomnia
Cannabidiol	5-HT1A partial agonists	Double-blind randomized placebo-controlled trial	Reduce subjective anxiety and increase mental sedation	No induce psychotic symptoms or mental disorders
Nefazodone trazodone and mirtazapine	5-HT2A receptor antagonist	Combined treatment with SSRIs Placebo-controlled trials	Enhance anti-depression and anti-anxiety of SSRI	Atypical antipsychotics should be prescribed with caution due to abundant evidence of side effects
Fluoxetine	Inhibitor of the serotonin transporter, 5-HT2B receptor partial agonist	Placebo-controlled randomized designs	In animal experiments, reduce abnormal behaviors in model mice	Further clinical experiments are needed
Agomelatine	5-HT2C receptor antagonist	Placebo controlled and active-comparator trials	Significant short-term and sustained antidepressant efficacy relative to placebo, venlafaxine and sertraline	Good acceptability and favorable safety profile
Ondansetron	5-HT3 receptor antagonist	Combined treatment with SSRIs using double blind placebo-controlled randomized designs	Augmented the antidepressant activity of the SSRI aroxetine has been shown to improve the mental state and social behavior of a schizophrenic patient	Have broad therapeutic window
SB-742457	5-HT6 antagonist	Placebo-controlled trials	Enhance cognition and attenuate anxiety and depression-like behaviors	Good tolerance, safety is similar to placebo
Amisulpride	Competitive antagonistic properties at 5-HT7 receptor	Randomized, placebo controlled trials	Effective drug for patients with MDD	Fewer extrapyramidal side-effects
Imipramine	Blocke histaminic, cholinergic, and alpha1-adrenergic receptor sites	Double-blind randomized placebo-controlled trial	At present, it is mainly used in the treatment of severe chronic depression. And the treatment effect of men is better than that of women	Gender differences in the adverse events caused by mipramine, with women more likely to report nausea and men more likely to report urinary difficulties and sexual dysfunction.
Desmethyl imipramine	Norepinephrine and serotonin reuptake inhibitor that works by blocking the reuptake of these neurotransmitters, thereby increasing their concentration in the synaptic cleft and improving the efficiency of neurotransmitter transport and transmission	Double-blind randomized placebo-controlled trial	It is primarily used to treat mild to moderate depression.	Dry mouth, constipation, dizziness, and blurred vision.
Vortioxetine	A SERT blocker, a 5-HT3, 5-HT7 receptor antagonist, and a 5-HT1A receptor agonist	Randomized clinical trials	Did not significantly improve GAD symptoms, QoL and functional status compared with a placebo treatment.	Safe and well tolerated
Venlafaxine	A mixed serotonin-norepine phrine reuptake inhibitor that binds and blocks both the SERT and NET transporters.	Randomized clinical trials	Offers no demonstrated advantages over SSRIs in terms of efficacy.	A higher risk of cardiovascular adverse effects and of fatal overdoses than with most SSRI antidepressants.
Sertraline	Inhibiting presynaptic reuptake of serotonin from the synaptic cleft.	Randomized controlled trials	Sertraline over some other antidepressants for the acute phase treatment of major depression was found, either in terms of efficacy (fluoxetine) or acceptability/tolerability (amitriptyline, imipramine, paroxetine and mirtazapine).	In terms of individual side effects, sertraline was generally associated with a higher rate of participants experiencing diarrhea.
Agmelatine	A melatonergic agonist and a 5-HT2C antagonist	Andomized, placebo controlled trials	The results of a large-scale clinical trial program, conducted in MDD, indicate both an antidepressant activity and a favorable tolerability profile.	Longer effect with fewer side effects on body weight, gastrointestinal tract, sexual function, and dizziness

### 3.2. 5-HT1B/1D, 5-HT1E, and 5-HT1F receptors

The 5-HT1B and 5-HT1A receptors share 43% of the homologous amino acid sequences. While 5-HT1A autoreceptors are located on the soma and dendrites, 5-HT1B/1D receptors are located on presynaptic axon terminals and act either as autoreceptors to control 5-HT release or heteroreceptors to regulate the release of other neurotransmitters. The 5-HT1B autoreceptor is an inhibitory GPCR. A lack of 5-HT1B autoreceptors in the hippocampus can reduce anxiety and depression-like behavior. In the bed nucleus of the stria terminalis, inhibition of 5-HT1B autoreceptors selectively weakens the anxiogenic effects of cocaine. Overexpression of 5-HT1B autoreceptors in the dorsal raphe nucleus of rats through viral-mediated delivery results in anti-anxiety, antidepressive, and reduced fear behavior. These differences may be due to cell-type specificity. Activation of 5-HT1B heteroreceptors can lead to antidepressant-like effects. The reduced expression of 5-HT1B heteroreceptors in the ventral striatum is associated with human depression and is believed to interact with p11 (a 5-HT1BR binding protein) to affect depressive behavior (Klein et al., 2017). The combination of 5-HT1B receptor antagonist GR-127935 or SB-216641 with imipramine, desmethylimipramine, or cobinamide had a significant anti-static effect on rats in a forced swimming test by disinhibiting 5-HT release by blocking presynaptic 5-HT1B autoreceptors (Carr and Lucki, 2011). The clinical significance of other 5-HT1 receptors (5-HT1E and 5-HT1F) remains unclear; however, the high expression of 5-HT1E receptors in the frontal cortex and hippocampus suggests a potential role in cognition. These receptors are widely distributed throughout the brain (Figure 2) (Karmakar and Lal, 2021).

### 3.3. 5-HT2 receptors

#### 3.3.1. 5-HT2A receptor

The 5-HT2A protomer, found in the 5-HT1AR-5-HT2AR heterocomplex, can reduce signaling via the Gi/o-AC-PKA pathway by inhibiting receptor–receptor interactions, which play a significant role in regulating emotions. Additionally, the OXTR (Oxytocin receptor)-5-HT2AR heteroreceptor complex promotes an increase in IP3 production and intracellular calcium release (Borroto-Escuela et al., 2021). 5-HT2A receptors are distributed in the forebrain, particularly the cortices and caudate nucleus (Figure 2). 5-HT2A receptors are postsynaptic receptors concentrated in the neocortex. Activation of the 5-HT2A receptor increased anxiety levels in a mouse model. In contrast, atypical antipsychotics, such as nefazodone, trazodone, and mirtazapine (Table 1), which are 5-HT2A receptor antagonists, enhanced the antidepressant and anxiolytic effects of SSRIs (Smits et al., 2008). Moreover, the activation of 5-HT2A receptors enhances glutamate release and participates in memory processes. Activation of the 5-HT2A receptor is thought to enhance glutamate release through the regulation of NMDA (N-methyl-D-aspartate) receptors, which play a critical role in synaptic plasticity and memory formation. This process occurs in both the neocortex and hippocampus. Activation of 5-HT2A receptors is believed to potentially underlie the antidepressant and anxiolytic effects of hallucinogenic drugs, which have been tested in recent clinical trials (Nautiyal and Hen, 2017). However, the mechanisms of 5-HT2A receptor activation by hallucinogenic drugs remain unclear. Therefore, it is crucial to identify the signal transduction pathways involved in this activation to better understand their potential in treating depression and anxiety. Overall, the role of 5-HT2A receptors in depression treatment is still controversial, and further research is needed to determine their precise mechanisms of action.

#### 3.3.2. 5-HT2B receptor

5-HT2B receptors are mainly located in the peripheral systems, such as the circulatory, respiratory, gastrointestinal, and urinary systems, with fewer receptors expressed in the CNS, in areas such as the nucleus of the nasal septum, dorsal hypothalamus, and medial amygdala (Figure 2). The peripheral localization of 5-HT2B receptors underlies the side effects of 5-HT-based therapies (Carr and Lucki, 2011). The 5-HT2B receptor, a member of the GPCR family of receptors, acts in essentially the same way as the 5-HT2A receptor and plays a role in the antidepressant effects of SSRIs (Fang et al., 2022). Fluoxetine is an SSRI that enhances the concentration of 5-HT in the brain (Table 1). By increasing 5-HT levels, fluoxetine improves neurogenesis and neuroplasticity, which are essential processes for learning, memory formation, and brain repair. Additionally, fluoxetine has anti-inflammatory properties that may contribute to its antidepressant effects by reducing neuroinflammation, which has been linked to the pathophysiology of depression (Surget, 2011). Fluoxetine also reduces depressive symptoms by restricting astrocyte activation through the 5-HT2B receptor/β-arrestin 2 pathway in a mouse model of major depressive disorder, which makes 5-HT2B a novel antidepressive target.

#### 3.3.3. 5-HT2C receptor

5-HT2C receptors are located in the limbic system, including the hippocampus, orthonasal olfactory system, and amygdala (Figure 2). An overexpression of 5-HT2C receptors was found in the prefrontal cortices of some suicide victims, which suggests a potential pathogenic role of 5-HT2C in depression and anxiety. Moreover, acute SSRI intake may precipitate anxiety through the activation of 5-HT1A autoreceptors and 5-HT2C receptors (Stamm et al., 2017; Hagsäter et al., 2019). Agomelatine is a novel antidepressant that functions as a 5-HT2C receptor antagonist and melatonin receptor agonist (MT1 and MT2) (Table 1). This dual mechanism of action provides both antidepressant and anxiolytic effects, making it an appealing option for treating depression. In comparison to placebos, venlafaxine, or sertraline, agomelatine has been demonstrated to have an earlier onset and long-lasting effect with fewer side effects, including less impact on body weight, the gastrointestinal tract, sexual function, and dizziness (Table 1). These advantages render agomelatine a promising alternative to traditional antidepressants for patients who may be sensitive to these common side effects (Olié and Kasper, 2007).

### 3.4. 5-HT3 receptor

5-HT3 receptors are distributed throughout the CNS, particularly in the spinal cord and brainstem (Figure 2). Cerebral 5-HT3 receptors participate in depression, anxiety, reward, pain, and the vomiting reflex (Bhatt et al., 2021). 5-HT3 receptors are ligand-gated ion channels that open transmembrane channels causing the inflow of extracellular calcium triggering neurotransmitter and/or polypeptide release. This rapid synaptic transmission capacity associates the 5-HT3 receptor with migraine, vomiting, drug addiction, neurodegenerative diseases, and a few neurological diseases. Moreover, 5-HT3 receptors in the dorsal vagal complex are involved in controlling vomiting and serve as the targets of 5-HT3 receptor antagonists, such as ondansetron, tropisetron, and dolasetron, which were developed as antiemetics for chemotherapy-induced vomiting (Bhatt et al., 2021) (Table 1).

### 3.5. 5-HT4 receptor

In the CNS, 5-HT4 receptors are found in the limbic system including the amygdala, septal area, hippocampus, and mesolimbic circuit (Tanaka et al., 2012). In the periphery, 5-HT4 receptors are distributed in the cardiovascular, gastrointestinal, urinary, and endocrine systems (Figure 2). Activation of 5-HT4 receptors triggers the intracellular cAMP (cyclic adenosine monophosphate) signaling pathway through adenylate cyclase activation and is involved in synaptic plasticity and memory (Bhatt et al., 2021). The binding capacities of 5-HT4 receptors and cAMP levels were decreased in multiple brain regions of suicide victims with depression and violence (Karayol et al., 2021). Furthermore, the lower striatal binding capacity of 5-HT4 receptors has been reported as a possible contributing factor to major depressive disorder (Madsen et al., 2014). These studies suggest a role for 5-HT4 receptors in psychiatric disorders, and that activation of 5-HT4 receptors is a potential therapeutic mechanism for depression and anxiety (Carr and Lucki, 2011; Köhler-Forsberg et al., 2022).

### 3.6. 5-HT5 receptor

5-HT5 receptors have been identified in the hippocampus, cerebral cortex, amyloid nucleus, and raphe nucleus and are involved in learning and memory (Figure 2). SB-6995516 (a 5-HT5A receptor antagonist) was shown to improve the associative learning task of autoshaping, highlighting this receptor's critical role in cognitive processing (Liu et al., 2020). This effect is most likely attributed to the crucial role that the receptor plays in memory formation, as both its activation and blockade have been demonstrated to impact memory processes. Further research is necessary to fully understand the underlying mechanisms and the specific situations in which these effects become apparent, as well as to clarify the distinct 5-HT5 receptor subtypes and their diverse modulators.

### 3.7. 5-HT6 receptor

5-HT6 receptors are postsynaptic GPCRs located in the corpus striatum, nucleus accumbens, cerebral cortex, amygdala, hippocampus, and hypothalamus (Figure 2) (Quintero-Villegas and Valdés-Ferrer, 2022). Recent studies have demonstrated the antidepressant and anxiolytic effects of two specific 5-HT6 receptor agonists in rodents (Carr et al., 2011). The 5-HT6 receptor agonists enhanced the effects of haloperidol and reduced the haloperidol-induced side effects (Wesołowska et al., 2021). Furthermore, 5-HT6 receptor antagonists such as SB-399885 have been shown to have antidepressant effects in animal studies. For instance, the combination of SB-399885 with imipramine, desipramine, bupropion, and clofibride enhanced the anti-static effects in the forced swimming test of rats, suggesting an antidepressant effect of 5-HT6 receptor blockade (Wesołowska, 2010; Carr and Lucki, 2011) (Table 1). Paradoxical results are explained by the specific modulation of cerebral neurocircuits by different 5-HT6 receptor agonists or antagonists. Further studies are needed to clarify the brain region specificity of 5-HT6 receptors and their implication in depression (Wesołowska et al., 2021).

### 3.8. 5-HT7 receptor

5-HT7 receptors were recently discovered and are mainly located in the thalamus, hypothalamus, hippocampus, and cortex (Figure 2). The 5-HT7 receptor belongs to the GPCR family (Bonaventure et al., 2012) and acts through the adenylyl cyclase-cAMP pathway (Gasbarri and Pompili, 2014). Studies have confirmed that 5-HT7 receptors regulate sleep, circadian rhythms, emotions, sensations, and gastrointestinal mobility. Amisulpride, an atypical antipsychotic, is a D2/D3 receptor(a group of dopamine receptors) antagonist that acts as an antidepressant by competitively inhibiting 5-HT7 receptors (Table 1). In mice devoid of the 5-HT7 receptor, the antidepressant-like effect of amisulpride was eliminated (Hołuj et al., 2015). Therefore, 5-HT7 receptor antagonists are considered promising therapeutic modalities for depression.

## 4. Summary and outlook

Numerous studies have confirmed the role of the serotonergic system in the pathophysiology of depression and anxiety, which identified 5-HT and its receptors as the main therapeutic targets. SSRIs are the most efficient and tolerable antidepressants. Unfortunately, 70% of the patients that respond to SSRIs are not completely treated, and the remaining patients are resistant to treatment. The serotonergic system is complex, owing to the autoreceptor and heteroreceptor organization of specific 5-HT receptor subtypes. Therefore, identifying the underlying mechanisms and specific signal transduction in neural circuits underlying depression and anxiety could pave the way for producing ideal therapeutic drugs with greater efficacy and tolerability (Slifirski et al., 2021).

## Author contributions

DC and YL are responsible for ensuring that the descriptions are accurate, agreed upon by all authors, and were responsible for developing the idea for the original draft, writing guidance, and submission. JG, JC, and WL were responsible for writing the original draft, literature search, and English editing. JL, LW, and RS were responsible for the original draft's content and structure. All authors have contributed to the manuscript and approved the submitted version.

## References

[B1] Albert P. R. Vahid-Ansari F. (2019). The 5-HT1A receptor: Signaling to behavior. Biochimie. 161:34–45. 10.1016/j.biochi.2018.10.015 31079617

[B2] Barnes N. M. Ahren G. P. Becamel C. Bockaert J. Camilleri M. Dubel S.. (2021). International union of basic and clinical pharmacology. cx. classification of receptors for 5-hydroxytryptamine; pharmacology and function. Pharmacol Rev 73(1): 310–520. 10.1124/pr.118.015552 33370241 PMC7770494

[B3] Bhatt S. Devadoss T. Manjula S. N. Rajangam J. (2021). 5-HT3 receptor antagonism a potential therapeutic approach for the treatment of depression and other disorders. Curr. Neuropharmacol. 19, 1545–1559. 10.2174/1570159X18666201015155816 33059577 PMC8762176

[B4] Bonaventure P. Dugovic C. Kramer M. De Boer P. Singh J. Wilson S.. (2012). Translational evaluation of JNJ-18038683, a 5-hydroxytryptamine type 7 receptor antagonist, on rapid eye movement sleep and in major depressive disorder. J. Pharmacol. Exp. Ther. 342, 429–440. 10.1124/jpet.112.193995 22570363

[B5] Borroto-Escuela D. O. Ambrogini P. Chruścicka B. Lindskog M. Crespo-Ramirez M. Hernández-Mondragón J. C.. (2021). The role of central serotonin neurons and 5-ht heteroreceptor complexes in the pathophysiology of depression: a historical perspective and future prospects. Int. J. Mol. Sci. 22, 4. 10.3390/ijms22041927 33672070 PMC7919680

[B6] Browne C. A. Jacobson M. L. Lucki I. (2020). Novel targets to treat depression: opioid-based therapeutics. Harv. Rev. Psychiatry. 28, 40–59. 10.1097/HRP.0000000000000242 31913981

[B7] Calhoun C. A. Lattouf C. Lewis V. Barrientos H. Donaldson S. T. (2023). Chronic mild stress induces differential depression-like symptoms and c-Fos and 5HT1A protein levels in high-anxiety female Long Evans rats. Behav. Brain Res. 438, 114202. 10.1016/j.bbr.2022.114202 36343695 PMC9990717

[B8] Carr G. V. Lucki I. (2011). The role of serotonin receptor subtypes in treating depression: a review of animal studies. Psychopharmacol. (Berl.). 213, 265–287. 10.1007/s00213-010-2097-z 21107537 PMC3374933

[B9] Carr G. V. Schechter L. E. Lucki I. (2011). Antidepressant and anxiolytic effects of selective 5-HT6 receptor agonists in rats. Psychopharmacol. (Berl.). 213, 499–507. 10.1007/s00213-010-1798-7 20217056 PMC2910165

[B10] Cipriani A. Furukawa T. A. Salanti G. Chaimani A. Atkinson L. Z. Ogawa Y.. (2018). Comparative efficacy and acceptability of 21 antidepressant drugs for the acute treatment of adults with major depressive disorder: a systematic review and network meta-analysis. Lancet. 391, 1357–1366. 10.1016/S0140-6736(17)32802-7 29477251 PMC5889788

[B11] Cipriani A. Zhou X. Del Giovane C. Hetrick S. E. Qin B. Whittington C.. (2016). Comparative efficacy and tolerability of antidepressants for major depressive disorder in children and adolescents: a network meta-analysis. Lancet. 388, 881–890. 10.1016/S0140-6736(16)30385-3 27289172

[B12] Davey C. G. Chanen A. M. Hetrick S. E. Cotton S. M. Ratheesh A. Amminger G. P.. (2019). The addition of fluoxetine to cognitive behavioural therapy for youth depression (YoDA-C): a randomised, double-blind, placebo-controlled, multicentre clinical trial. Lancet Psychiatry. 6, 735–744. 10.1016/S2215-0366(19)30215-9 31371212

[B13] Fang Y. Ding X. Zhang Y. Cai L. Ge Y. Ma K.. (2022). Fluoxetine inhibited the activation of A1 reactive astrocyte in a mouse model of major depressive disorder through astrocytic 5-HT2BR/β-arrestin2 pathway. J. Neuroinflammation. 19, 23. 10.1186/s12974-022-02389-y 35093099 PMC8800238

[B14] García-Gutiérrez M. S. Navarrete F. Gasparyan A. Austrich-Olivares A. Sala F. Manzanares J. (2020). Cannabidiol: a potential new alternative for the treatment of anxiety, depression, and psychotic disorders. Biomolecules. 10. 10.3390/biom10111575 33228239 PMC7699613

[B15] Gasbarri A. Pompili A. (2014). Serotonergic 5-HT7 receptors and cognition. Rev. Neurosci. 25, 311–323. 10.1515/revneuro-2013-0066 24486730

[B16] Gelenberg A. J. (2010). A review of the current guidelines for depression treatment. J. Clin. Psychiatry. 71, e15. 10.4088/JCP.9078tx1c 20667285

[B17] Guzel T. Mirowska-Guzel D. (2022). The role of serotonin neurotransmission in gastrointestinal tract and pharmacotherapy. Molecules. 27. 10.3390/molecules27051680 35268781 PMC8911970

[B18] Hagsäter S. M. Thorén J. Pettersson R. Eriksson E. (2019). Selective serotonin reuptake inhibition increases noise burst-induced unconditioned and context-conditioned freezing. Acta Neuropsychiatr. 31, 46–51. 10.1017/neu.2018.26 30404671

[B19] Healy D. Le Noury J. Jureidini J. (2019). Paediatric antidepressants: benefits and risks. Int. J. Risk Saf. Med. 30, 1–7. 10.3233/JRS-180746 29865094

[B20] Hołuj M. Popik P. Nikiforuk A. (2015). Improvement of ketamine-induced social withdrawal in rats: the role of 5-HT7 receptors. Behav. Pharmacol. 26, 766–775. 10.1097/FBP.0000000000000132 25769091

[B21] Karayol R. Medrihan L. Warner-Schmidt J. L. Fait B. W. Rao M. N. Holzner E. B.. (2021). Serotonin receptor 4 in the hippocampus modulates mood and anxiety. Mol. Psychiatry. 26, 2334–2349. 10.1038/s41380-020-00994-y 33441982 PMC8275670

[B22] Karmakar S. Lal G. (2021). Role of serotonin receptor signaling in cancer cells and anti-tumor immunity. Theranostics 11, 5296–5312. 10.7150/thno.55986 33859748 PMC8039959

[B23] Klein A. K. Brito M. Akhavan S. Flangen D. Le N. Ohana T.. (2017). Attenuation of the anxiogenic effects of cocaine by 5-HT(1B) autoreceptor stimulation in the bed nucleus of the stria terminalis of rats. Psychopharmacology (Berl). 234, 485–495. 10.1007/s00213-016-4479-3 27888284 PMC5226880

[B24] Köhler-Forsberg K. Ozenne B. Larsen S. V. Poulsen A. S. Landman E. B. Dam V. H.. (2022). Concurrent anxiety in patients with major depression and cerebral serotonin 4 receptor binding. A NeuroPharm-1 study. Transl. Psychiatry. 12, 273. 10.1038/s41398-022-02034-5 35821015 PMC9276803

[B25] Lanfumey L. Hamon M. (2004). 5-HT1 receptors. Curr. Drug Targets CNS Neurol. Disord. 3, 1–10. 10.2174/1568007043482570 14965240

[B26] Liu Q. Q. Yao X. X. Gao S. H. Li R. Li B. J. Yang W.. (2020). Role of 5-HT receptors in neuropathic pain: potential therapeutic implications. Pharmacol. Res. 159, 104949. 10.1016/j.phrs.2020.104949 32464329

[B27] Madsen K Torstensen E. Holst K. K. Haahr M. E. Knorr U. Frokjaer V. G. (2014)(1). Familial risk for major depression is associated with lower striatal 5-HT4 receptor binding. Int. J. Neuropsychopharmacol. 18. 10.1093/ijnp/pyu034 25522384 PMC4368872

[B28] Mamdani F. Berlim M. T. Beaulieu M. M. Turecki G. (2014). Pharmacogenomic predictors of citalopram treatment outcome in major depressive disorder. World J. Biol. Psychiatry. 15, 135–144. 10.3109/15622975.2013.766762 23530732 PMC5293541

[B29] Martiny K. (2017). Novel augmentation strategies in major depression. Dan. Med. J. 64, B5338. 28385173

[B30] Mendez-David I. David D. J. Darcet F. Wu M. V. Kerdine-Römer S. Gardier A. M.. (2014). Rapid anxiolytic effects of a 5-HT4 receptor agonist are mediated by a neurogenesis-independent mechanism. Neuropsychopharmacology. 39, 1366–1378. 10.1038/npp.2013.332 24287720 PMC3988540

[B31] Muit J. J. van Eijndhoven P. F. P. Cipriani A. Dalhuisen I. van Bronswijk S. Furukawa T. A.. (2022). Efficacy and acceptability of next step treatment strategies in adults with treatment-resistant major depressive disorder: protocol for systematic review and network meta-analysis. BMJ Open. 12, e056777. 10.1136/bmjopen-2021-056777 35437250 PMC9016400

[B32] Nautiyal K. M. Hen R. (2017). Serotonin receptors in depression: from A to B. F1000Res. 6, 123. 10.12688/f1000research.9736.1 28232871 PMC5302148

[B33] Nichols D. E. Nichols C. D. (2008). Serotonin receptors. Chem. Rev. 108, 1614–1641. 10.1021/cr078224o 18476671

[B34] Olié J. P. Kasper S. (2007). Efficacy of agomelatine, a MT1/MT2 receptor agonist with 5-HT2C antagonistic properties, in major depressive disorder. Int. J. Neuropsychopharmacol. 10, 661–673. 10.1017/S1461145707007766 17477888

[B35] Quintero-Villegas A. Valdés-Ferrer S. I. (2022). Central nervous system effects of 5-HT7 receptors: a potential target for neurodegenerative diseases. Mol. Med. 28, 70. 10.1186/s10020-022-00497-2 35725396 PMC9208181

[B36] Razakarivony O. Newman-Tancredi A. Zimmer L. (2021). Towards in vivo imaging of functionally active 5-HT1A receptors in schizophrenia: concepts and challenges. Transl. Psychiatry. 11, 22. 10.1038/s41398-020-01119-3 33414418 PMC7791062

[B37] Samuels B. A. Nautiyal K. M. Kruegel A. C. Levinstein M. R. Magalong V. M. Gassaway M. M.. (2017). The behavioral effects of the antidepressant tianeptine require the mu-opioid receptor. Neuropsychopharmacology. 42, 2052–2063. 10.1038/npp.2017.60 28303899 PMC5561344

[B38] Slifirski G. Król M. Turło J. (2021). 5-HT receptors and the development of new antidepressants. *Int. J. Mol. Sci*. 22. 10.3390/ijms22169015 34445721 PMC8396477

[B39] Smits K. M. Smits L. J. Peeters F. P. Schouten J. S. Janssen R. G. Smeets H. J.. (2008). The influence of 5-HTTLPR and STin2 polymorphisms in the serotonin transporter gene on treatment effect of selective serotonin reuptake inhibitors in depressive patients. Psychiatr. Genet. 18, 184–190. 10.1097/YPG.0b013e3283050aca 18628680

[B40] Stamm S. Gruber S. B. Rabchevsky A. G. Emeson R. B. (2017). The activity of the serotonin receptor 2C is regulated by alternative splicing. Hum. Genet. 136, 1079–1091. 10.1007/s00439-017-1826-3 28664341 PMC5873585

[B41] Surget A Tanti A Leonardo E. D Laugeray A Rainer Q Touma C. (2011). Antidepressants recruit new neurons to improve stress response regulation. Mol. Psychiatry. 16, 1177–1188. 10.1038/mp.2011.48 21537331 PMC3223314

[B42] Tanaka K. F. Samuels B. A. Hen R. (2012). Serotonin receptor expression along the dorsal-ventral axis of mouse hippocampus. Philos. Trans. R. Soc. Lond. B Biol. Sci. 367, 2395–2401. 10.1098/rstb.2012.0038 22826340 PMC3405677

[B43] Wang Q Dong X Hu T Qu C Lu J Zhou Y. (2021). Constitutive activity of serotonin receptor 6 regulates human cerebral organoids formation and depression-like behaviors. Stem Cell Rep. 16, 75–88. 10.1016/j.stemcr.2020.11.015 33357407 PMC7815944

[B44] Wesołowska A. (2010). Potential role of the 5-HT6 receptor in depression and anxiety: an overview of preclinical data. Pharmacol. Rep. 62, 564–577. 10.1016/S1734-1140(10)70315-7 20884998

[B45] Wesołowska A. Rychtyk J. Gdula-Argasińska J. Górecka K. Wilczyńska-Zawal N. Jastrzebska-Wiesek M.. (2021). Effect of 5-HT6 receptor ligands combined with haloperidol or risperidone on antidepressant-/anxiolytic-like behavior and BDNF regulation in hippocampus and prefrontal cortex of rats. Neuropsychiatr. Dis. Treat. 17, 2105–2127. 10.2147/NDT.S309818 34211274 PMC8240864

[B46] Yohn C. N. Gergues M. M. Samuels B. A. (2017). The role of 5-HT receptors in depression. Mol. Brain. 10, 28. 10.1186/s13041-017-0306-y 28646910 PMC5483313

